# Supplementary material for: Characterization of Plasma-Derived Small Extracellular Vesicles Indicates Ongoing Endothelial and Platelet Activation in Patients with Thrombotic Antiphospholipid Syndrome
Source: Cells. 2020 May 13;9(5):1211. doi: 10.3390/cells9051211 (PMC7290474; doi:10.3390/cells9051211)
Supplement: Supplementary file 1 [file cells-09-01211-s001.pdf]

Supplementary Table S1. List of 37 markers used in the MACSPlex Exosome Kit and their cellular expression.

Marker	Aliases	Cellular expression
CD1c	BDCA-1	Major population of myeloid DCs
CD2	LFA-2	Thymocytes, NK cells, T- and B-cells
CD3	CD3d molecule	Mature T-cells, thymocytes, subset of NK cells
CD4	CD4 molecule	Thymocyte subsets, T helper cells, regulatory T cells, monocytes, macrophages
CD8	CD8 molecule	Thymocyte subsets, cytotoxic T cells, subset of NK cells, myeloid DC
CD9	TSPAN29, P24, MIC3	sEVs marker<br>Platelets, pre-B-cells, eosinophils, basophils, activated T-cells, endothelial and epithelial cells
CD11c	ITGAX	Monocytes, macrophages, granulocytes, NK cells, myeloid DCs, subset of B- and T-cell
CD14	CD14 molecule	Monocytes, macrophages, subset of neutrophils and myeloid DCs, Langerhans cells, and granulocytes
CD19	CD19 molecule	B-cells and follicular DC
CD20	MS4A1	T- and B-cell subsets
CD24	Heat stable antigen (HSA)	B-cells, granulocytes, epithelial cells, monocytes, neutrophils, neuroblasts
CD25	IL2RA	Activated T- and B-cells, some thymocytes, regulatory T cells, oligodendrocytes, and macrophages
CD29	ITGB1	Leukocytes
CD31	PECAM-1	Monocytes, platelets, granulocytes, endothelial cells, lymphocyte subsets, and epithelial cells
CD40	CD40 molecule	B-cells, monocytes, macrophages, follicular DC, endothelial cells, fibroblasts, and keratinocytes
CD41b	ITGA2B	Megakaryocytes and platelets
CD42a	GP9	Megakaryocytes and platelets
CD44	CD44 molecule	Cancer stem cells, hematopoietic, fibroblastic, and glial cells
CD45	Leukocyte common antigen, PTPRC	Hematopoietic cells (except erythrocytes and platelets)
CD49e	ITGA5	Thymocytes, T-cells, early activated B-cells, monocytes, platelets, fibroblasts, endothelial, and epithelial cells
CD56	NCAM1	NK cells and T-cell subsets
CD62P	SELP	Activated endothelial cells, platelets and megakaryocytes
CD63	LAMP-3, MLA1	sEVs marker<br>Activated platelets, monocytes, macrophages, granulocytes, and endothelial cells
CD69	CD69 molecule	Activated leukocytes, NK cells, thymocyte subsets, platelets, Langerhans cells, and activated macrophages
CD81	TAPA1, Tetraspanin-28	sEVs marker<br>B- and T-cells, NK cells, monocytes, thymocytes, DCs, endothelial cells, and fibroblasts
CD86	CD86 molecule	Activated T- and B-cells, monocytes, DCs, and endothelial cells
CD105	ENG	Mature endothelial cells, mesenchymal stem cells, erythroid precursors, activated monocytes and macrophages
CD133/1	PROM1	Multipotent progenitor cells; including immature hematopoietic stem and progenitor cells, circulation endothelial progenitor cells, fetal neural stem cells, other tissue-specific stem cells, and cancer stem cells
CD142	F3; TF	Activated endothelial cells, epithelial cells, monocytes, macrophages, platelets, astrocytes, and Schwann cells
CD146	MCAM, MUC18, Mel-CAM	Endothelial cells, pericytes, smooth muscle cells, follicular DC, melanoma cells, subpopulation of activated T-cells, marrow stromal cells (MSCs)
CD209	DC-SIGN	DC, placental macrophages, endothelial cells of placental vascular channels, peripheral blood mononuclear cells
CD326	EpCAM	Embryonic stem cells, epithelial cells in tissues, some circulating tumor cells, and cancer stem cells
HLA-ABC		APC

HLA-DRDPDQ		APC
MCSP	CSPG4	Some cancer cells
ROR1	ROR1	Some cancer cells
SSEA-4	Sialyl Lewis x, CD15	Stem cell marker

Supplementary table 1 adapted from Koliha, 2016 [32] and https://www.abcam.com/primary-antibodies/human-cd-antigen-guide

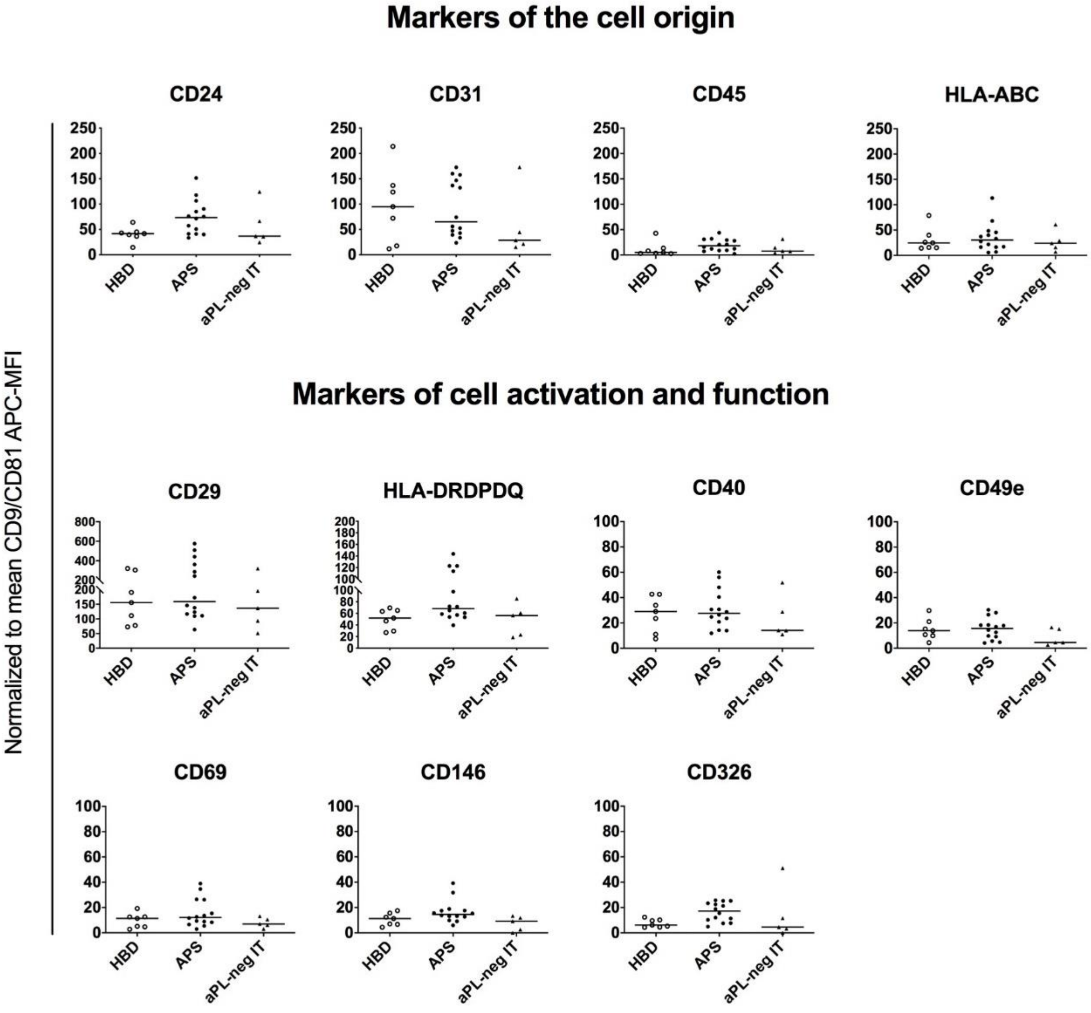

Supplementary Figure S1. Normalized median fluorescence intensities (MFI) of the surface protein profiles of the plasma-derived sEVs from healthy blood donors (HBD), patients with antiphospholipid syndrome (APS) and aPL-neg patients with idiopathic thrombosis (aPL-neg IT). The nonparametric Kruskal–Wallis test with Dunn's multiple comparison adjustment was used. Grouped surface protein profiles are shown indicating the cell of origin (upper panel) and cell activation status/functional properties of the sEVs (lower panel).
